# User profile of an online cognitive behavioral therapy self-help platform in Turkey

**DOI:** 10.1007/s12144-023-04787-8

**Published:** 2023-05-31

**Authors:** Ömer Özer, Aydoğan Aykut Ceyhan, Sascha Y. Struijs

**Affiliations:** 1 Open Education Faculty, Department of Social Work and Consultancy, Anadolu University, Eskisehir, Turkey; 2 Department of Clinical, Neuro and Developmental Psychology, Vrije Universiteit Amsterdam, Amsterdam, the Netherlands; 3 Education Faculty, Department Guidance and Psychological Counseling, Anadolu University, Eskisehir, Turkey

**Keywords:** Internet based intervention, User characteristics, Self help, Pandemic

## Abstract

Online mental health self-help services are of societal importance and increasingly popular. Therefore, we have developed an online platform offering free self-help to the Turkish public with modules based on Cognitive Behavioral Therapy (CBT) targeting depression, anxiety, and stress respectively. The main purpose of this study is to describe the user profile of this platform. A pre-intervention self-report assessment including general demographic information and the Brief Symptom Inventory questionnaire during October 2020 until September 2022. 8331 participants completed the assessment and created an account out of the 11.228 users who registered during a two-year period, of which 8.331 (74%) completed the assessment and created an account. The majority of these users were female (76.17%), highly educated (82%), single (68%) and actively studying or working (84%). Slightly more than half (57%) of the platform user had not received psychological assistance before, while those who did receive previous assistance indicate to have benefitted from that (74%). The psychological symptoms of users are widely distributed, encompassing a broad range of user profiles. Approximately half of all users actively used the platform, while the other half did not complete any module. Among active users, the course “coping with depressive mood” was the most popular (41.45%), followed by “coping with anxiety” (37.25%) and “coping with stress” (21.30%). Offering a free online CBT self-help platform to the Turkish public seems feasible, with strong uptake among both man and woman struggling with a variety of psychological symptoms. Further research is needed to assess user satisfaction and change in symptoms over time during platform use by means of a feasibility trial.

Past natural disasters and economic crises affecting large groups, including the COVID-19 pandemic, have had negative impacts on the mental health of people (Makwana, 2019). Indeed, studies conducted in various countries indicate that symptoms of depression, anxiety, sleep problems, and post-traumatic stress disorder increase during the pandemic/epidemic(Alharbi et al., 2022; Chevance et al., 2020; González-Sanguino et al., 2020; Pierce et al., 2020; Tang et al., 2020). In addition, the systematic review study evaluating the results of 43 studies reveals that the post-traumatic stress symptoms and depression levels of individuals infected with COVID-19 virus disease increased, the symptoms of individuals with a previous diagnosis of a psychiatric disorder worsened, especially the psychological symptoms of healthcare professionals increased, and the general psychological well-being level of the society decreased (Vindegaard & Benros, 2020). In a systematic review of studies evaluating the impact of quarantine measures taken during the pandemic on psychological symptoms, it was found that anxiety, anger, and post-traumatic stress symptoms increased during this period (Brooks et al., 2020). It was also reported that symptoms differed in specific groups such as cancer patients and women before and after COVID-19 (Ayaz et al., 2020; Han et al., 2021). In addition to the increase in psychological symptoms during the pandemic, changes that directly affect the individual’s life, such as quarantine measures and economic problems, made it difficult for individuals to access mental health services. In this process, the need for mental health assistance has also increased for most individuals. In a survey conducted by the World Health Organization in 130 countries in six regions between June and August 2022, it was emphasized that there were 67% disruptions in counseling and psychotherapy services, 70% disruptions in the access of elderly individuals to services, and up to 35% disruptions in emergency interventions required for reasons such as delirium, prolonged seizures, and substance withdrawal syndrome (World Health Organization, 2022). This has revealed the necessity of reevaluating the existing interventions in the delivery of mental health services after the COVID-19 pandemic and developing new applications in the future. In this context, web-based self-help systems have come to the fore as an important means of delivering mental health services to large masses.

Self-help systems can be used as unguided systems without any expert support, as a part of the face-to-face intervention and as an independent intervention method, and with the developing technology (Cuijpers & Schuurmans, 2007), they are often offered through mobile applications or web pages. In the literature, the effectiveness of web-based self-help applications has been proven in numerous problem areas such as depressive mood and anxiety problems (Cuijpers et al., 2019; Karyotaki et al., 2021; Kenter et al., 2016; Lewis et al., 2012; van Straten et al., 2008), stress management (Amanvermez et al., 2021; Richardson & Rothstein, 2008; Ugalde et al., 2017), eating disorders (Aardoom et al., 2016; Hamatani et al., 2022; Moghimi et al., 2021), addictions such as alcohol, gambling, and substance addiction (Baumgartner et al., 2019; Riper et al., 2011; Schaub et al., 2019, 2021), and obsessive-compulsive disorders(Dèttore et al., 2015; Imai et al., 2022; Rachamim et al., 2022). While the effectiveness of web-based self-help applications has been demonstrated in many studies, users of these applications often remain unknown. In this respect, studies investigating the user profile of self-help systems are of great importance.

In a study in the literature, user characteristics in the pilot implementation of PUSH-D (Mehrotra et al., 2017), an intervention program for depressive mood in India, show that the program is predominantly used by females, individuals under the age of 50, mostly graduates, and those with higher education. In another study, it was reported that 97% of the users of the Break Binge Eating platform, which was developed to intervene in eating disorders, were female, 64% had not received psychological assistance before, and 88% were not currently receiving any help for eating disorders (Linardon et al., 2020). In another application developed to prevent depression, despite being a preventive website, it is stated that only 30% of the users have symptoms at this level, the rest show high levels of depression, and similarly, the users of this system were primarily female(Muñoz et al., 2021). The MindSpot application, which is actively used in the Australian health system, is reported to be also predominantly used by women (73.4%), the age range of the users is 18–93 years with an average age of 35.4 years, and the prevalence of suicidal thoughts is 30%, and the rate of depressive mood is 70.5% (Titov et al., 2020). These findings in the literature indicate that self-help platform users were generally female, individuals with high symptom levels, and individuals with previous experience of seeking psychological assistance.

Studies on Internet-based interventions used in the field of mental health in Turkey are limited. There are mostly review studies on this subject (Acar, 2022; Çetintulum-Huyut, 2019; Durdu Akgün et al., 2019). In addition, there are two studies evaluating the effects of Internet-based intervention on cognitive errors and psychological symptoms in children (Buğa & Hamamcı, 2016) and the effect of Internet-based intervention on patients with obsessive-compulsive disorder (Göcek-Yorulmaz, 2020) in small study groups. Apart from these, there is no study on a web-based intervention and its users and effectiveness in this regard. The preliminary study (Özer & Ceyhan, 2021), which introduces and describes the general features of the self-help platform developed to help individuals cope with depressive mood, anxiety, and stress-related symptoms during COVID-19, is the first large-scale study in this field in Turkey. Therefore, we want to reveal the user profiles and identify factors associated with engagement of this platform. This will be an important indicator for future studies. We expect users to be young, highly educated and mostly female, as these demographics are associated with the use of online self-help in previous studies (Linardon et al., 2020; Mehrotra et al., 2017; Muñoz et al., 2021; Titov et al., 2020).

Self-help interventions, which are also called public open online interventions, should be seen as an important opportunity for increasing and scaling up access to psychological help services today (Muñoz et al., 2016). Indeed, revealing who uses these interventions will make important contributions to the development of content suitable for the user profile. It will also help the development of systems that are sensitive to the needs and usage preferences of individuals in need of psychological assistance services and may pave the way for the necessary arrangements to make existing systems user-friendly. The main purpose of this study is to describe the user profile of the self-help system, open to the public, developed in Turkey during the pandemic related to COVID-19 disease. In this context, taking into account the gender of the users, (1) their general characteristics, (2) their previous psychological assistance status, (3) their COVID-19 experiences, (4) their psychological symptom levels, and (5) their use of the platform content were revealed.

## Method

### Participants

The number of users registered to the self-help system as of September 2022 was 11,228. Of these users, 2897 (28.80%) did not complete the measurement tools, in other words, they did not log in to the system after registering and filling out the required measurement tools. The number of self-help platform users who registered to the system and completed the measurement tools was 8331, and the current study group consists of these participants. Of the users, 1985 (23.8%) were male, and 6346 (76.17%) were female. The ages of the users ranged from 18 to 68, with an average age of 28.25 (s.8.54).

### Measures

#### Self-help platform user Registration Form

The form was prepared by the researchers to obtain information about the general information of the users (gender, education level, marital status, employment status, and income level), their previous psychological assistance status (type of assistance, and the most recent assistance received), and their COVID-19 experiences (whether they or anyone in their family has had COVID-19, whether they have experienced COVID-related loss).

#### Brief Symptom Inventory

The inventory was developed by Derogatis to measure the psychological symptoms of individuals and adapted to Turkish. The four-point Likert-type inventory consists of 53 items. The items address the symptoms of the respondent based on the last two weeks. The measurement instrument consists of nine sub-scales, three global indices, and additional items. The sub-scales include somatization, obsessive-compulsive disorder, interpersonal sensitivity, depression, anxiety, hostility, phobic anxiety, paranoid thoughts, and psychoticism. The global indices include the global severity index, symptom total, and symptom distress index. The inventory can be used by taking into account different sub-scale structures, and in this study, depression, anxiety, somatization, negative self and hostility sub-scales, and total symptom score were used. In the adaptation process of the inventory, four different studies were conducted and it was found that the internal consistency coefficients of the sub-scales of the inventory ranged between 0.63 and 0.86, and the internal consistency coefficients of the total score ranged between 0.93 and 0.96 (Hisli Sahin & Durak, 1994). In this study, the internal consistency coefficients of the psychological symptom sub-scales were 0.88 for anxiety, 0.90 for depression, 0.89 for negative self, 0.79 for hostility, 0.81 for somatization, and 0.96 for the total scale.

### Data Collection

Within the scope of the study, data were collected online through the platform available at http://kendikendineyardim.anadolu.edu.tr. The self-help platform consists of programs for coping with depressed mood, anxiety, and stress. Each program consists of five modules. All modules are structured based on cognitive behavioral therapy techniques. The names and contents of the modules are shown in Table 1.

**Table 1:** Modules in the Self-Help Platform Programs*

	Coping with Depressed Mood	Coping with Anxiety	Coping with Stress
Module 1	What is depressed mood?	What is anxiety?	What is stress?
Module 2	Behavioral activation	Our world of thought	Progressive muscle relaxation
Module 3	Our world of thought	Challenging automatic thoughts	Emotion-thought behavior relationship
Module 4	Working with thoughts	Behavior experiment	Stress time
Module 5	Coping with repetitive thoughts	Facing anxieties	Problem solving

* Detailed information about the relevant platform can be obtained from http://kendikendineyardim.anadolu.edu.tr platform address and Özer and Ceyhan (2021)

The module contents given in Table 1 were developed by researchers working in the fields of psychology, psychiatry, psychological counseling and guidance, and experienced in cognitive behavioral therapy. The development of the platform started right at the beginning of the pandemic and was made available in October 2020. Moreover, printed materials of the content on the platform are available, and users can provide self-help services using printed materials as well as online content. Online access to the platform is still open to all users.

The data of the study includes the data of the users who logged in to the platform for the first time between October 2020, which is the opening date of the platform, and October 2022, created a membership, provided informed consent, and responded to the relevant measurement tools. On the platform, new users first complete their membership by entering their e-mail, age, gender, and city, and then they are asked to fill out the Self-Help Member Registration Form and the Brief Symptom Inventory. Users cannot access the platform content without completing the relevant measurement tools. In this context, data were obtained from 8331 users who had registered as users and completed the measurement tools in a period of approximately two years. The data obtained were analyzed using descriptive statistics within the scope of this study. Ethical approval was obtained from Anadolu University Social and Humanities Science Scientific Research and Publication Ethics Committee (24.06.2020/35,755).

## Results

Within the scope of the study, taking into account the gender of the self-help platform users, their (1) general characteristics, (2) previous psychological assistance status, (3) COVID-19 experiences, (4) psychological symptom levels, and (5) use of platform content were investigated.

1. Findings on General Information of the users

The distribution of platform users according to their gender, education level, marital status, and employment status is presented in Table 2.

**Table 2:** General Characteristics of the Users by Gender

Characteristics		Male		Female		Total	
		**n**	**%**	**n**	**%**	**n**	**%**
**Gender**		1985	23.83	6346	76.17	8331	100
Education Level	High School	257	12.95	566	8.92	823	9.88
	University Student	806	40.61	2927	46.12	3733	44.81
	University Graduate	737	37.13	2353	37.08	3090	37.09
	Postgraduate	185	9.32	500	7.88	685	8.22
Marital Status	Married	651	32.80	1729	27.25	2380	28.57
	Single	1277	64.33	4395	69.26	5672	68.08
	Divorced	57	2.87	222	3.50	279	3.35
Employment Status	Student	735	37.03	3182	50.14	3917	47.02
	Unemployed/Seeking jobs	192	9.67	976	15.38	1168	14.02
	Retired	47	2.37	77	1.21	124	1.49
	Employed	1011	50.93	2111	33.27	3122	37.47

As shown in Table 2, the majority of the users were female (n = 6346, 76.17%), and female users are approximately three times more than male users. In terms of education level, the majority of users were university students and university graduates for both males and females, and these two education levels account for approximately 82% of all users. Likewise, a significant portion of both male and female users was single (68%), and the majority were students and employed individuals. Students and employees constitute approximately 74% of all users.

2. Findings related to previous psychological assistance status of users

In the study, the experiences of platform users seeking psychological assistance were investigated according to their gender and whether they had received psychological assistance before, the form/type of psychological assistance they had received previously, and the time of the last psychological assistance received. The findings obtained are shown in Table 3.

**Table 3:** Previous Psychological Assistance Status of the Users

		Male		Female		Total	
		N	%	n	%	n	%
Previous Psychological Assistance Status	Yes	800	40.30	2804	44.19	3604	43.26
	No	1185	59.70	3548	55.91	4733	56.81
Type of Assistance	Individual therapy/counseling	595	74.38	2063	73.57	2658	73.75
	Medication treatment	196	24.50	719	25.64	915	25.39
	Other (family, friend support)	9	1.13	22	0.78	31	0.86
Time of last assistance received	In the last 6 months	507	63.38	1564	55.78	2071	57.46
	6 months − 1 year ago	71	8.88	312	11.13	383	10.63
	1–5 years ago	147	18.38	592	21.11	739	20.50
	5 + years ago	75	9.38	336	11.98	411	11.40

As shown in Table 3, it is noteworthy that slightly more than half (59.70% and 55.91%) of the platform users, both men, and women, have not received psychological assistance services before. As for both male and female users with psychological assistance experience, it is seen that the majority or about two-thirds of them (73.75% of the total number of users who received help) benefited from individual counseling processes. Likewise, in terms of the last time users received assistance, approximately a little more than half of the users (57.46%) have had a psychological assistance experience in the last six months, and in terms of gender, although the rate of females receiving psychological assistance in the last six months is slightly higher than that of males, a similar pattern is seen when the last five year time periods are considered.

3. Findings regarding COVID-19 Experiences of the users

The COVID-19 experiences of the users according to their gender were investigated in terms of whether they had COVID, whether at least one of their family members had COVID, and whether they had experienced loss due to COVID, and the findings were presented in Table 4.

**Table 4:** COVID-19 Experiences of the Users

		Male (N = 1985)		Female (N = 6346)		Total (N = 8331)	
		N	%	N	%	N	%
Status of contracting COVID-19	Yes	338	17.03	1093	17.22	1431	17.18
	No	1647	82.97	5253	82.78	6900	82.82
Having at least one family member who had COVID-19	Yes	624	31.44	2178	34.32	2802	33.63
	No	1361	68.56	4168	65.68	5529	66.37
Loss of family member due to COVID-19	Yes	31	1.56	121	1.91	152	1.82
	No	1954	98.44	6225	98.09	6803	98.17

Considering the COVID experiences in Table 4, it is seen that while 17.18% of the users have had COVID-19, 82.82% have not. Therefore, it is seen that approximately four-fifths of all users have not been infected with the COVID-19 virus, and this pattern is the same in terms of gender. In terms of whether any of the users’ family members have had COVID-19, it is noteworthy that about one-third (33.61%) of the users have had a family member who has had COVID-19, and about two-thirds (66.37%) of the users do not have any family member who has had such an experience, and this rate was quite similar in terms of gender. In terms of the loss of a family member due to COVID-19, 98.17% did not have such a loss and this has a similar pattern in terms of gender.

4. Findings on psychological symptom levels of the users

The psychological symptom levels of the platform users were examined in terms of depression, anxiety, somatization, hostility, and negative self sub-scales of the Brief Symptom Inventory and total/general symptom score in terms of gender. Information on the psychological symptom levels of the users is presented in Table 5.

**Table 5:** Psychological Symptom Levels of the Users

		x̄	Median	S	Min	Max
Psychological Symptoms Total	Male	73.51	69.00	39.13	0	206
	Female	88.00	87.00	40.17	0	207
Depression	Male	21.20	20.00	11.28	0	48
	Female	25.23	26.00	10.96	0	48
Anxiety	Male	16.79	15.00	10.29	0	52
	Female	20.43	19.00	10.85	0	52
Somatization	Male	7.39	6.00	5.87	0	36
	Female	10.11	9.00	6.65	0	34
Hostility	Male	10.39	10.00	5.69	0	28
	Female	11.60	11.00	5.70	0	28
Negative Self	Male	17.75	17.00	10.83	0	48
	Female	20.63	20.00	11.20	0	48

* Male n = 1985, Female n = 6346

** The lowest and highest scores of the scale: 0-212 for total psychological symptoms, 0–48 for the depression sub-scale, 0–52 for the anxiety sub-scale, 0–36 for the somatization sub-scale, 0–28 for the hostility sub-scale, and 0–48 for the negative self sub-scale

As seen in Table 5, when the psychological symptom levels of the users are taken into consideration, it is seen that the mean scores of the total symptom score and the mean scores of the sub-scales such as depression, somatization, hostility, and negative self are slightly higher in females than in males. In addition, when the maximum and minimum score range of the users in both the total psychological symptoms and sub-scales are examined, it is seen that both male and female users show a wide distribution that reflects the entire range of scores that can be obtained from the scale. Therefore, it is seen that the platform users include a wide user profile that includes all levels of psychological symptoms.

5. Findings on Engagement of users in platform content

Out of a total of 8331 users who registered to use the platform, 4027 users (48.34%) completed the modules in the program in varying numbers, while 4303 users (51.66%) terminated their login session in the system without completing any module related to the program content. The findings regarding the completion of the content of the modules in the program after logging into the system are shown in Table 6.

**Table 6:** Use of the Self-Help Platform by the Users

	Program	Number of Completed Modules											
		1 module		2 modules		3 modules		4 modules		5 modules		Total	
		n	%	n	%	n	%	n	%	n	%	n	%
Male	Coping with Depressed Mood	115	11.92%	121	12.54%	80	8.29%	18	1.87%	21	2.18%	355	36.79%
	Coping with Anxiety	105	10.88%	107	11.09%	87	9.02%	30	3.11%	33	3.42%	362	37.51%
	Coping with Stress	78	8.08%	67	6.94%	73	7.57%	16	1.66%	14	1.45%	248	25.7%
	Total	298	30.88%	295	30.57%	240	24.87%	64	6.63%	68	7.05%	965	100.00%
Female	Coping with Depressed Mood	375	12.25%	452	14.76%	314	10.26%	74	2.42%	99	3.23%	1314	42.91%
	Coping with Anxiety	322	10.52%	366	11.95%	262	8.56%	69	2.25%	119	3.89%	1138	37.17%
	Coping with Stress	190	6.21%	163	5.32%	175	5.72%	40	1.31%	42	1.37%	610	19.92%
	Total	887	28.97%	981	32.04%	751	24.53%	183	5.98%	260	8.49%	3062	100.00%
TOTAL	Coping with Depressed Mood	490	12.17%	573	14.23%	394	9.78%	92	2.29%	120	2.98%	1669	41.45%
	Coping with Anxiety	427	10.60%	473	11.75%	349	8.67%	99	2.46%	152	3.78%	1500	37.25%
	Coping with Stress	268	6.66%	230	5.71%	248	6.16%	56	1.39%	56	1.39%	858	21.31%
	Total	1185	29.43%	1276	31.69%	991	24.61%	247	6.13%	328	8.15%	4027	100.00%

As can be seen in Table 6, when the usage status of the programs on the platform after user registration is examined, it is seen that users completed the modules in the programs for coping with depressive mood (41.45%), coping with anxiety (37.25%), and coping with stress (21.30%), respectively. When this is examined in terms of gender, it is seen that while this order remains the same for women, it changes slightly for men and that the highest usage for men is in the module on coping with anxiety, followed closely by the program on coping with depressive mood. When the number of completion of the modules in all three programs is examined, it is noteworthy that the usage numbers of the modules in all three programs have gradually decreased. This decrease is observed to be the same for both men and women. In addition, the completion rates of a total of five modules in each of the three programs were similar, ranging from 1.37 to 3.88%.

## Discussion and conclusion

In this study, the user profile of an Internet-based self-help platform consisting of depressive mood, anxiety, and stress-coping modules was evaluated. In this context, the general characteristics of the users, COVID-19 experiences, previous psychological assistance status, psychological symptoms, and system usage characteristics were evaluated. The study has the largest study group compared to the applications developed in Turkey. In this respect, it can be stated that it reveals important data on the user profile.

There is no consensus in the literature on who uses Internet-based interventions. Individuals who are older, female, separated/divorced, and have a graduate degree, as well as individuals with a current or past history of depression, higher levels of depressive symptoms, and lower internal stigma are more likely to use these interventions (Crisp & Griffiths, 2014). The users of the SH platform are also predominantly women. It is seen that there are similar findings in open access systems on this subject. In Australia, 93% of the users of a web-based intervention developed for eating disorders were females (Linardon et al., 2020). In another intervention program on depressive mood in Mexico, the rate of male users was 15.6% (Lara et al., 2014). In another intervention on trauma, 86% of the participants were female (Vona et al., 2014). Meta-analysis studies also emphasize that users are mostly females (Davies et al., 2014). In the long-term evaluation (2013–2019) of the Internet-based intervention used as a public health intervention in Australia, it is stated that the rate of female users ranged between 71.6% and 74.3% for seven years. This may be related to the help-seeking attitudes of individuals. It is frequently stated in the literature that women’s help-seeking volunteerism is higher than men’s. Similar findings were also observed in Turkey (Odaci & Kalkan, 2005; Serim & Cihangir Çankaya, 2015). Although they are recommended as a method for young men to receive psychological assistance, Internet-based interventions are mostly preferred by women, which may be related to gender roles. There is a need for more research on the attitudes and usage patterns of male users toward Internet-based interventions.

In the study, the majority of the users were university students and university graduates (82%), singles (68%), and students and employed individuals (74%). These rates were quite similar in both the male and female groups. These findings suggest that platform users are generally young adults. One reason for this may be that the system on which the platform is located has a university domain name, and individuals from all age groups were not sufficiently informed about using the system during the pandemic. Another issue is that during the pandemic, universities have largely switched to distance education, digital natives (young people who are more comfortable using technology) may have been more likely to seek help on self-help platforms in the digital space, given that their developmental, particularly social needs were quite limited during the pandemic period. Indeed, in support of these explanations, it is seen that slightly more than half of the platform users (59.70% and 55.91%), both males and females, have not received psychological assistance services before.

When COVID experiences are taken into consideration, it is seen that the majority of users have not contracted the COVID-19 virus (82.82%), have not had such a loss in terms of losing a family member due to COVID-19 (98.17%), and this has a similar pattern in terms of gender. This result suggests that the conditions and limitations of the pandemic are an important factor in users’ seeking psychological assistance rather than the experience of COVID-19-related illness. Indeed, in support of this view, when the usage status of the programs on the platform is examined, it is seen that users completed the modules on coping with depressive mood (41.45%), coping with anxiety (37.25%), and coping with stress (21.30%), respectively.

Symptom levels of users are another variable addressed within the scope of the study. When the averages of the study evaluating the validity of the Brief Symptom Inventory in Turkish young people and the averages of the platform users are compared, it is seen that individuals using the SH platform have higher mean psychological symptoms in all sub-scales (Hisli Sahin et al., 2002). The mean psychological symptom scores of individuals who were admitted to the university counseling and guidance center to receive psychological assistance were 24.45 for depression (mean values of the platform users were 21.2 for males, 25.23 for females), 18.78 for anxiety (males 16.79, females 20.43), 11.63 for hostility (males 10.39, females 11.6) 9.4 for negative self (males 17.75, females 20.63), 6.77 for somatization (males 7.39, females 10.1), and 82.39 for total score (males 73.51, females 88.0) (Ozer & Altinok, 2015). From this point of view, it can be stated that the mean psychological symptom averages of the SH platform users are higher than the general population and close to the mean scores of the population applying for face-to-face psychological assistance. Another study evaluating the user profile over seven years emphasizes that the self-help system was used by users with high symptom levels (Titov et al., 2020). It is also stated that people with high levels of psychological distress are more prone to use applications (Powell et al., 2003; Ryan et al., 2010). In this context, it can be stated that the user profile in Turkey is similar to the profiles in other countries. In future studies, self-help applications can be tested on clinical samples, and the effectiveness of using the intervention platform for symptom intensity can be evaluated in terms of outcomes at the end of the intervention.

Another striking finding in the current study is the early dropout rates. Of the members of the self-help platform, 2897 (28.80%) only completed the short membership form and left the system without even filling out the measurement tools. Of the 8331 people who completed the surveys, 4304 (51.66%) did not start any module. It can be stated that almost one out of two people are members of the system but did not use the application. The completion rate of the group using the application was 8.14% (N = 328). Early dropout is one of the most important problem areas of Internet-based interventions. In their systematic review of early dropout in Internet-based interventions, Melville et al. (2010) report that early dropout in these programs varies between 2% and 83%, with an average of 31%. As in face-to-face psychological help processes, early dropout is a multifactorial process and is reported at different rates due to difficulties in identifying the dropouts (Wierzbicki & Pekarik, 1993). In some studies, not completing the whole system, and in others, the minimum number of sessions were considered indicators of early dropout. It can be stated that the likelihood of early dropout is lower among older users, so it is likely that the low average age of our study population, which is primarily university students, affects the rate of dropout (Karyotaki et al., 2015). In general, it can be stated that the early dropout rate in the study is consistent with the literature. However, studies on the predictors of early dropout in Internet-based interventions can contribute to the efficient use of these interventions. Gender, age, and the approach used may be determinants of dropout (Melville et al., 2010). Another reason for the high rates of early dropout is that the SH platform is an unguided intervention program. Early dropout rates in unguided intervention programs are higher than in guided programs (Boß et al., 2018; Jernelöv et al., 2012). In future studies, the factors predicting early dropout in Internet-based interventions and the reasons for an early dropout can be investigated. Moreover, early dropout rates in guided and unguided applications can also be compared in future studies.

As in all scientific studies, this study has some limitations. Firstly, the study design only allows for a descriptive analysis of the user profile and does not include any intervention or control group, so it is impossible to draw conclusions about the effectiveness of the online platform. Secondly, the study only includes self-reported data, which could be biased and may not accurately reflect the actual psychological symptoms or history of the users.

Next to these limitations, most of the participants have a university education or above. Therefore, the user profiles should also be explored in persons with different levels of education. Within the scope of this dissemination, it may be possible to obtain broader profiles of platform users to improve the interventions in the platform content. In this study, the usability and effectiveness of the program were not evaluated. However, the user profile here is considered valuable in terms of getting to know the current platform users and thus providing preliminary information for the development of the program. Therefore, it is considered that in the subsequent process, studies on the usability and effectiveness of the platform and the program can also be carried out when the program is completed by a greater number of participants in the future. In particular, the fact that a considerable number of users did not complete the program and quit early also reveals the need to examine the platform in terms of the acceptability of Internet-based interventions, user participation, and feasibility assessment.

Since the SH Platform is a first in its field, it should be considered a starting point for the development of Internet-based self-help interventions in Turkey. In the future, it is believed that addressing specific interventions specific to problem areas in a broader context and including new intervention programs based on different approaches besides cognitive behavioral therapy will make significant contributions to the provision of mental health services to a wide segment of society. In addition, it is believed that such platforms can make significant contributions to the effective use of mental health professionals to support professional psychological assistance services offered to individuals.

The findings of the study suggest that providing free online self-help modules based on Cognitive Behavioural Therapy may be an effective option for individuals experiencing psychological symptoms in Turkey. The popularity of the “coping with depressive mood” module suggests that depression is a major concern for those seeking mental health support. However, further research is required to determine user satisfaction and the effectiveness of the platform in reducing symptoms over time. Future research could explore users’ experiences in depth using quantitative research methods. We recommend that research using new data collection techniques like ecological momentary assesment EMA (Shiffman et al., 2008; Voogt et al., 2014) and online photovoice methods (Tanhan & Strack, 2020) be conducted to assess internet-based interventions.

## Data Availability

The datasets generated during and/or analyzed during the current study are available from the corresponding author on reasonable request.
